# A Multi-Verse Optimizer with Levy Flights for Numerical Optimization and Its Application in Test Scheduling for Network-on-Chip

**DOI:** 10.1371/journal.pone.0167341

**Published:** 2016-12-07

**Authors:** Cong Hu, Zhi Li, Tian Zhou, Aijun Zhu, Chuanpei Xu

**Affiliations:** 1 School of Mechano-Electronic Engineering, Xidian University, Xi'an, Shaanxi, China; 2 School of Electronic Engineering and Automation, Guilin University of Electronic Technology, Guilin, Guangxi, China; 3 Guilin University of Aerospace Technology, Guilin, Guangxi, China; West Virginia University, UNITED STATES

## Abstract

We propose a new meta-heuristic algorithm named Levy flights multi-verse optimizer (LFMVO), which incorporates Levy flights into multi-verse optimizer (MVO) algorithm to solve numerical and engineering optimization problems. The Original MVO easily falls into stagnation when wormholes stochastically re-span a number of universes (solutions) around the best universe achieved over the course of iterations. Since Levy flights are superior in exploring unknown, large-scale search space, they are integrated into the previous best universe to force MVO out of stagnation. We test this method on three sets of 23 well-known benchmark test functions and an NP complete problem of test scheduling for Network-on-Chip (NoC). Experimental results prove that the proposed LFMVO is more competitive than its peers in both the quality of the resulting solutions and convergence speed.

## Introduction

Optimization problems are common in the field of science and technology [1]. These problems are often nonlinear, multimodal or discontinuous, and very challenging to solve with traditional optimization methods. In the past few years, a number of meta-heuristic algorithms have been successfully developed to solve these problems. These techniques are inspired by natural phenomena or other sources using iterations and stochasticity to generate better solutions for optimization problems [2, 3]. Some popular meta-heuristic algorithms are the Genetic Algorithm (GA) [4], Ant Colony Optimization (ACO) [5], Particle Swarm Optimization (PSO) [6], Differential Evolution (DE) [7], Harmony Search (HS) [8], Artificial Bee Colony(ABC) [9], Cuckoo Search (CS) [10], Gravitational Search Algorithm (GSA) [11], Fruit Fly Optimization algorithm (FOA) [12], Gases Brownian Motion Optimization (GBMO) [13], Symbiotic Organisms Search (SOS) [14], and Moth-flame Optimization (MFO) [15].

Multi-verse optimizer (MVO) is a promising and up-to-date optimization algorithm proposed by [16]. As the name implies, it is inspired by the theory of multi-verse in physics. The three main concepts of the multi-verse theory (white hole, black hole, and wormhole) are the basis for the MVO algorithm. The concepts of white hole and black hole were utilized to explore search spaces by MVO. The wormholes help MVO exploit the search spaces. The MVO algorithm was first evaluated by 19 challenging test benchmarks. To further evaluate its performance, the MVO was adopted for five practical engineering problems. The experimental results prove that the proposed algorithm can produce very competitive results and outperform other algorithms described in the literature.

However, there are still some issues associated with this algorithm. When wormholes stochastically re-span a number of universes around the best universe achieved over the course of iterations, the MVO is likely to get trapped in the local optima.

To improve the global search ability of MVO and enhance the ability to escape from local optima, MVO is combined with Levy flights (LFMVO) in this paper. Levy flights, proposed by Paul Levy in 1937, are a type of random walk of generalized Brownian motion that include non-Gaussian randomly distributed step sizes for the distance moved. The Levy distribution features long tails, an infinite second order moment and convergence to a non-Gaussian stable distribution [17]. A number of natural and artificial events can be described by Levy flights, e.g., fluid dynamics, earthquake analysis, cooling behavior, diffusion of fluorescent molecules, noise and foraging paths (albatross, bumblebees, deer etc.) [18, 19].

Recently, Levy flights were added to nature-inspired algorithms to enhance their performance [18, 20–24]. In [20], Levy flights were adopted to generate new solutions (new cuckoo) in the cuckoo search. Since its step length is ultimately much longer, the strategy is more efficient for exploring the search space. In [21], the Levy-flight firefly algorithm (LFA) is introduced, which blends Levy flights with the search strategy to enhance the randomization of the firefly algorithm (FA). In [22], a Levy mutation is used in evolutionary algorithms because it is likely to create a new solution that is farther away from its parent solution than Gaussian mutation. Candela et al. [23] used Levy flights as a means to diversify ant colony optimization. Haklı et al. [18] presented a novel particle swarm optimization algorithm using Levy flight (LFPSO) in which a more efficient search occurs in the search space due to the long jumps executed by the particles. Therefore, the LFPSO is likely to avoid premature convergence and to improve the global search capability.

In this paper, the proposed method performs global search more effectively with random walks. If the universes cannot improve self-solutions, they are re-formed with Levy flights such that the best universe obtained so far is affected and being trapped in local optima is prevented. Experimental results with test benchmarks and test scheduling for NoC show the superiority of the LFMVO compared with the MVO algorithm and other algorithms.

This paper is organized as follows. The next section gives a brief overview of MVO. The following section presents a brief overview of Levy flights. The improved MVO algorithm called LFMVO algorithm is presented and analyzed in the LFMVO section. A comprehensive set of experimental results is provided in the Results section. An NP complete problem of test scheduling for NoC is presented in the Application section. Finally, the conclusions are drawn in last section.

## Brief overview of a multi-verse optimizer

### Multi-verse theory

Multi-verse theory is a new and well-known theory in physics. This theory implies the existence of universes beyond the one in which we live [25].

### Multi-verse optimizer

The concepts of white hole and black hole were utilized to explore search spaces by MVO. Wormholes help MVO exploit the search spaces. In MVO, a solution corresponds to a universe, a variable in the solution corresponds to an object in the universe, the inflation rate of a solution corresponds to the fitness of the solution, and the term time corresponds to the iteration.

A universe with a higher inflation rate is highly probable to have white holes and tend to send objects through white holes, whereas a universe with a lower inflation rate is highly probable to have black holes and tends to receive objects through black holes. The white/black hole tunnels are used to exchange objects between different universes. Despite the inflation rate, objects in all universes have a high probability of shifting to the best universe via wormholes.

A roulette wheel selection (RWS) is adopted to mathematically model the exchange of objects between universes and the white/black hole tunnels. At each iteration, one of the universes is chosen by the RWS to have a white hole based on its inflation rate.

Assume that

$$U=\left[\begin{array}{cccc} x_{11} & x_{12} & \cdots & x_{1d}\\ x_{21} & x_{22} & \cdots & x_{2d}\\ \vdots & \vdots & \vdots & \vdots \\ x_{n1} & x_{n2} & \cdots & x_{nd} \end{array}\right]$$ (1)

where *d* indicates the number of parameters (variables) and *n* denotes the number of universes (candidate solutions):

$$x_{ij}=\{\begin{array}{c} x_{kj}\;r1<NI(U_{i})\\ x_{ij}\;r1\geq NI(U_{i}) \end{array}$$ (2)

where *x*_*ij*_ expresses the jth parameter of the *i*th universe, *x*_*kj*_ expresses the *j*th parameter of the *k*th universe selected by an RWS, r1 ∈ [0, 1] denotes a random number, *U*_*i*_ denotes the *i*th universe, and *NI(U*_*i*_*)* denotes a normalized inflation rate of the *i*th universe.

The wormhole tunnels are always built between a universe and the best universe constituted so far to provide local changes for each universe and the high probability of refining the inflation rate via wormholes as follows:

$$x_{ij}=\{\begin{array}{l} \{\begin{array}{c} X_{j}+TDR\times ((ub_{j}-lb_{j})\times r4+lb_{j})\;r3<0.5\\ X_{j}-TDR\times ((ub_{j}-lb_{j})\times r4+lb_{j})\;r3\geq 0.5 \end{array}\;r2<WEP\\ \;x_{ij}\;r2\geq WEP \end{array}$$ (3)

where *X*_*j*_ is the *j*th parameter of the best universe constituted so far; travelling distance rate (*TDR*) and wormhole existence probability (*WEP*) are coefficient; *ub*_*j*_ and *lb*_*j*_ are the upper bound and the lower bound of *j*th variable, respectively; *x*_*ij*_ denotes the *j*th parameter of the *i*th universe; and *r2*, *r3*and *r4* are random numbers in [0, 1].

WEP is defined as the existence probability of wormholes in universes. To enhance exploitation during the progress of the optimization process, it increases linearly over the iterations.

$$WEP=Wmin+l\times (\frac{Wmax-Wmin}{L})$$ (4)

where *Wmin* indicates the minimum (commonly set to 0.2), *Wmax* indicates the maximum (commonly set to 1), *l* is the current iteration, and *L* is the maximum iteration.

TDR is defined as the distance rate by which an object can be teleported by a wormhole around the best universe obtained so far. To gain more precise exploitation/local search around the best universe, TDR is increased over the iterations.

$$TDR=1-\frac{l^{1/p}}{L^{1/p}}$$ (5)

where *p* (set to 6 in this paper) indicates the exploitation accuracy over the iterations.

The general steps of the MVO algorithm are described as follows. The optimization process starts by creating a set of random universes. At each iteration, objects in the universes with high inflation rates incline to shift to the universes with low inflation rates through white/black holes. Simultaneously, objects in each universe have the chance to randomly teleport to the best universe via wormholes. This process continues until it is terminated by satisfying an end criterion (e.g., maximum iterations).

## Brief overview of Levy flights

In general terms, Levy flights are a random walk whose step length obeys the Levy distribution. The Levy distribution is often in accordance with a simple power-law formula *L*(*s*) ∼ |s|^−1−*β*^, where 0 < *β* ≤ 2 is an index. Mathematically a simple version of the Levy distribution can be described as [18, 20]

$$L(s,\gamma ,\mu )=\{\begin{array}{l} \sqrt{\frac{\gamma }{2\pi }}\exp\left[-\frac{\gamma }{2(s-\mu )}\right]\frac{1}{{\left(s-\mu \right)}^{3/2}},\;0<\mu <s<\infty \\ \;0\;,\;s\leq 0 \end{array}$$ (6)

where *μ* denotes a location or shift parameter and *γ* > 0 denotes a scale parameter.

According to Fourier transform, a Levy distribution can be defined

$$F(k)=\exp[-\alpha {\left|k\right|}^{\beta }],\;0<\beta \leq 2$$ (7)

where *α* indicates skewness or scale factor and *β* indicates Levy index. The inverse of this integral does not have an analytical form for the general *β* except for a few special cases.

For the case of *β* = 2, we have

$$F(k)=\exp[-\alpha {\left|k\right|}^{2}]$$ (8)

whose inverse Fourier transform corresponds to a Gaussian distribution.

For the case of *β* = 1, we have

F(k)=exp[−α|k|] (9)

which corresponds to a Cauchy distribution

$$p(x,\gamma ,\mu )=\frac{1}{\pi }\frac{\gamma }{\gamma^{2}+{\left(x-\mu \right)}^{2}}$$ (10)

where *μ* is the location parameter and *γ* is the scale parameter.

For the general case, the inverse integral

$$L(s)=\frac{1}{\pi }\int_{0}^{\infty }\cos(ks)\exp[-\alpha {\left|k\right|}^{\beta }]dk$$ (11)

can be evaluated only when *s* is large enough. We have

$$L(s)\to \frac{\alpha \beta \Gamma (\beta )\sin(\pi \beta /2)}{\pi {\left|s\right|}^{1+\beta }},\;s\to \infty$$ (12)

where Γ(*z*) expresses the Gamma function

$$\Gamma (z)=\int_{0}^{\infty }m^{z-1}e^{-m}dm$$ (13)

When *z* = *n* is an integer, we have

Γ(n)=(n−1)! (14)

For exploring unknown, large-scale search spaces, Levy flights are superior to Brownian random walks [20, 26].

## The proposed LFMVO algorithm

In the original MVO algorithm, when wormholes stochastically re-span a number of the solution universes around the best solution achieved over the course of iterations, the MVO is likely to get trapped in the local optima.

If the universes cannot improve self-solutions, they are re-formed with Levy flights such that the best universe obtained so far is affected and being trapped in local optima is prevented.

In the proposed method, when generating new solutions $U_{i}^{t+1}$ (for universe *i*), a Levy flight is executed

$$U_{i}^{t+1}=U_{i}^{t}+K\times (Lb+(Ub\text{-}Lb)*Levy(x))\times U_{i}^{t}$$ (15)

where *K* is the Levy weight that controls the impact of the previous universe on the current universe, *Lb* is the lower bound of the feasible region, and *Ub* is the upper bound of the feasible region. It should be noted that a larger Levy weight inclines to facilitate a global search, while a smaller Levy weight to facilitate a local search. Therefore, the Levy weight *K* is crucial to the convergence behavior of MVO. A suitable value for the Levy weight usually provides a balance between global exploration and local exploitation and results in refined solutions. To achieve a trade-off between exploration and exploitation and to accelerate convergence speed, we proposed a Levy weight that linearly decreases over the course of iterations. In the early stages, a relatively large Levy weight is adopted to coarse-tune the whole search area. At the end stages, a relatively small Levy weight is adopted to fine-tune the current search area. This adaptive Levy weight factor (ALWF) is determined as follows.

K=(Max_Iter-t)/Max_Iter (16)

where *Max_Iter* is the maximum iterations, *t* is the current iteration.

It is not trivial to generate step size *s* samples using Levy flights [27]. There are several approaches to achieve step size samples, but the direct and efficient approach is to adopt the Mantegna algorithm [28]. In Mantegna's algorithm, the step size *s* can be described by

$$s=\frac{u}{{\left|v\right|}^{\frac{1}{\beta }}}$$ (17)

where *u* and *v* are drawn from normal distributions. That is

$$u∼N(0,\sigma^{2}),\;v=N(0,\sigma_{v}^{2})$$ (18)

with

$$\sigma ={\left(\frac{\Gamma (1+\beta )\times \sin(\frac{\pi \beta }{2})}{\Gamma (\frac{1+\beta }{2})\times \beta \times 2^{\left(\frac{\beta -1}{2}\right)}}\right)}^{\frac{1}{\beta }},\;\sigma_{v}=1$$ (19)

Thus, a simple scheme can be depicted as

$$Levy(x)=0.01\times \frac{u\times \sigma }{{\left|v\right|}^{\frac{1}{\beta }}}$$ (20)

where *β* is a constant (= 1.5) and *σ* is measured in Eq (19).

Based on the above, the pseudo code of the LFMVO is shown in Algorithm 1.

**Algorithm 1**: LFMVO algorithm

**Input:** NI (objective function)

d (number of variables)

n (number of universes)

Lb = [Lb_1_,Lb_2_,…,Lb_d_] (the lower bound of feasible region)

Ub = [Ub_1_,Ub_2_,…,Ub_d_] (the upper bound of feasible region)

*Max_Iter* (maximum number of iterations)

**Output:** The optimal objective function value *NI(BU)* and the optimal solution *BU*.

***Step1*: *Initialization***

Create random universes U using Eq (21)

*Initialize WER*, *TDR*, *and BU*

t = 0

***Step2*: *Sorting and Normalization***

SU = Sorted universes

NI = Normalize the inflation rate of the universes

***Step 3*:*Iteration***

***while*** *t<Max_Iter*

  *Evaluate the $NI(U_{i}^{t})$, i = 1,2,…,n*

  ***for*** *each universe Ui*

    *Update WEP and TDR using Eq (4) and Eq (5)*

    *BHI = i;*

    *Update U using Eq (15)*

    ***for*** *each object x*_*ij*_

      *r1 = rand (0*,*1);*

      ***if*** *r1<NI(Ui)*

      *WHI = RWS(-NI);*

      *U(BHI*,*j) = SU(WHI*,*j);*

      ***end if***

      *r2 = rand (0*,*1);*

      ***if*** *r2< WEP*

        *r3 = rand (0*,*1);*

        *r4 = rand (0*,*1);*

        *if r3<0*.*5*

            *x*_*ij*_ = BU(j) + TDR * ((Ub(j)- Lb(j)) * r4 + Lb(j));

        ***else***

            xij = BU(j)—TDR * ((Ub(j)-Lb(j)) * r4 + Lb(j));

        ***end if***

      ***end if***

    ***end for***

  ***end for***

  *t = t+1*

***end while***

***Step 4*: *Termination***

**Output** *BU* and *NI(BU)*

In step 1, the universes are randomly generated in a feasible region using Eq (23) for a given optimization problem. Let *UP* represent the universe population, which can be denoted as follows:

$$UP=\left\{U_{1},U_{2},\ldots ,U_{i},\ldots ,U_{n}\right\}$$ (21)

where *n* is the number of universes and *i* = 1, 2,…, n. Each universe *U*_*i*_ can be expressed as

$$U_{i}=(x_{i1},x_{i2},\ldots ,x_{ij},\ldots ,x_{id})$$ (22)

where *d* is the number of variables and *j* = 1, 2,…, d.

$$x_{ij}=Lb_{j}+(Ub_{j}-Lb_{j})\times rand(0,1)$$ (23)

where *Lb*_*j*_ is the lower bound of the *j*th variable, *Ub*_*j*_ is the upper bound of the *j*th variable, and *rand(0*, *1)* represents a random number in [0, 1].

In step 2, we sort the universe population into a non-decreasing order and normalize the inflation rate (fitness) of the universes.

Step 3 is the process of iterative optimization. First, we evaluate the fitness of all universes *NI(U*_*i*_*)* using Eq (13). Then, for each universe *U*_*i*_, we update WEP and TDR using Eq (4) and Eq (5), respectively. Next, we record the black hole index BHI and update the universes U using Eq (15). After that, we update each object *x*_*ij*_ of the universes using Eq (2) and Eq (3).

In step 4,when the end criterion is satisfied, the optimal objective function value *NI(BU)* and the optimal solution *BU* are obtained.

## Experimental Results and Discussion

To evaluate the performance of the proposed LFMVO algorithm, 23 standard benchmark functions are employed. These functions are well-known and have been widely adopted by many researchers. The functions are shown in Table 1, where *d* is the dimension of the function and *f*_*min*_ represents the optimum value of the function. The optimum values of functions f1-f13 are zero except for f8 which has an optimum value of -418.9829*d.All the functions f14-f23 have nonzero optimum values. The benchmark functions can be divided into three groups: unimodal benchmark functions (f1-f7), multi-modal benchmark functions (f8-f13), and fixed-dimension multimodal benchmark functions (f14-f23). The unimodal benchmark functions have one global optimum. However, the multi-modal test functions have a global optimum, and the number of local optima increases exponentially with the dimensions. The fixed-dimension multimodal benchmark functions have only a few local optima.

**Table 1:** The benchmark functions used in our experiments.

Test function	n	Range	f_min_
$f_{1}(x)=\sum_{i=1}^{d}x_{i}^{2}$	40	[-100,100]	0
$f_{2}(x)=\sum_{i=1}^{d}\left|x_{i}\right|+\prod_{i=1}^{d}\left|x_{i}\right|$	40	[-10,10]	0
$f_{3}(x)={\sum_{i=1}^{d}\left(\sum_{j-1}^{i}x_{j}\right)}^{2}$	40	[-100,100]	0
*f*_4_(*x*) = max_*i*_ {|*x*_*i*_|,1 ≤ *i* ≤ *d*}	40	[-100,100]	0
$f_{5}(x)=\sum_{i=1}^{d-1}\left[100{\left(x_{i+1}-x_{i}^{2}\right)}^{2}+{\left(x_{i}-1\right)}^{2}\right]$	40	[-30,30]	0
$f_{6}(x)={\sum_{i=1}^{d}\left[(x_{i}+0.5)\right]}^{2}$	40	[-100,100]	0
$f_{7}(x)=\sum_{i=1}^{d}ix_{i}^{4}+random[0,1)$	40	[-1.28,1.28]	0
$f_{8}(x)=\sum_{i=1}^{d}-x_{i}\sin(\sqrt{\left|x_{i}\right|})$	40	[-500,500]	-418.9829*d
$f_{9}(x)=\sum_{i=1}^{d}\left[x_{i}^{2}-10\cos(2\pi x_{i})+10\right]$	40	[-5.12,5.12]	0
$f_{10}(x)=-20\exp\left(-0.2\sqrt{\frac{1}{n}\sum_{i=1}^{d}x_{i}^{2}}\right)-\exp\left(\frac{1}{n}\sum_{i=1}^{d}\cos(2\pi x_{i})\right)+20+e$	40	[-32,32]	0
$f_{11}(x)=\frac{1}{4000}\sum_{i=1}^{d}x_{i}^{2}-\prod_{i=1}^{d}\cos\left(\frac{x_{i}}{\sqrt{i}}\right)+1$	40	[-600,600]	0
$\begin{array}{l} f_{12}(x)=\frac{\pi }{d}\left\{10\sin(\pi y_{1})+\sum_{i=1}^{d}{\left(y_{i}-1\right)}^{2}[1+10\sin^{2}(\pi y_{i+1})]+{\left(y_{d}-1\right)}^{2}\right\}\\ +\sum_{i=1}^{d}u(x_{i},10,100,4) \end{array}$ $y_{i}=1+\frac{x_{i}+1}{4}$, $u(x_{i},a,k,m)=\{\begin{array}{c} k{\left(x_{i}-a\right)}^{m},\;x_{i}>a\\ 0,\;-a<x_{i}<a\\ k{\left(-x_{i}-a\right)}^{m},\;x_{i}<-a \end{array}$	40	[-50,50]	0
$\begin{array}{l} f_{13}(x)=0.1\{\sin^{2}(3\pi x_{1})+\\ \sum_{i=1}^{d}{\left(x_{i}-1\right)}^{2}[1+\sin^{2}(3\pi x_{i}+1)]+{\left(x_{d}-1\right)}^{2}[1+\sin^{2}(2\pi x_{d})]\}\\ +\sum_{i=1}^{d}u(x_{i},5,100,4) \end{array}$	40	[-50,50]	0
$f_{14}(x)=\left(\frac{1}{500}\sum_{j=1}^{25}\frac{1}{j+\sum_{i=1}^{2}{\left(x_{i}-a_{ij}\right)}^{6}}\right)$	2	[-65.53,65.53]	0.998004
$f_{15}(x)={\sum_{i=1}^{11}\left[ai-\frac{x_{1}(b_{i}^{2}+b_{i}x_{2})}{b_{i}^{2}+b_{i}x_{3}+x_{4}}\right]}^{2}$	4	[-5,5]	0.0003075
$f_{16}(x)=4x_{1}^{2}-2.1x_{1}^{4}+\frac{1}{3}x_{1}^{6}+x_{1}x_{2}-4x_{2}^{2}+4x_{2}^{4}$	2	[-50,50]	-1.0316285
$f_{17}(x)={\left(x_{2}-\frac{5.1}{4\pi^{2}}x_{1}^{2}+\frac{5}{\pi }x_{1}-6\right)}^{2}+10(1-\frac{1}{8\pi })\cos x_{1}+10$	2	[-5,10]*[0,15]	0.398
$\begin{array}{l} f_{18}(x)=[1+{\left(x_{1}+x_{2}+1\right)}^{2}(19-14x_{1}+3x_{1}^{2}-14x_{2}+6x_{1}x_{2}+3x_{2}^{2})]\times \\ \left[30+{\left(2x_{1}-3x_{2}\right)}^{2}\times (18-32x_{1}+12x_{1}^{2}+48x_{2}-36x_{1}x_{2}+27x_{2}^{2})\right] \end{array}$	2	[-5,5]	3
$f_{19}(x)=-\sum_{i=1}^{4}c_{i}\;\exp(-\sum_{j=1}^{3}a_{ij}{\left(x_{j}-p_{ij}\right)}^{2})$	3	[-0,1]	-3.86
$f_{20}(x)=-\sum_{i=1}^{4}c_{i}\;\exp(-\sum_{j=1}^{6}a_{ij}{\left(x_{j}-p_{ij}\right)}^{2})$	6	[-0,1]	-3.32
$f_{21}(x)=-{\sum_{i=1}^{5}\left[(X-a_{i}){\left(X-a_{i}\right)}^{T}+c_{i}\right]}^{-1}$	4	[0,10]	-10.1532
$f_{22}(x)=-{\sum_{i=1}^{7}\left[(X-a_{i}){\left(X-a_{i}\right)}^{T}+c_{i}\right]}^{-1}$	4	[0,10]	-10.4029
$f_{23}(x)=-{\sum_{i=1}^{10}\left[(X-a_{i}){\left(X-a_{i}\right)}^{T}+c_{i}\right]}^{-1}$	4	[0,10]	-10.5364

We set the dimension of the test functions (f1-f13) to 40. To have a fair comparison, all algorithms have the same population size (set to 60) and the same maximum number of iterations (set to 600). We run each algorithm 40 times so that we can execute significant statistical analysis (e.g., best, mean and standard deviation). The parameter settings of the algorithms, which are commonly used in the literature, are provided in Table 2. For verification of the results, we compare the LFMVO algorithm with MVO, PSO and MFO, as shown in Tables 3–5.

**Table 2:** The parameter settings of the algorithms.

Algorithm	Tuning Parameter	Value
LFMVO	WEP_Max	1
	WEP_Min	0.2
	p (Exploitation accuracy)	6
	*β* (Levy index)	1.5
MVO [16]	Wmax (max WEP)	1
	Wmin (min WEP)	0.2
	p (Exploitation accuracy)	6
PSO [29]	c1 (Cognitive constant)	2
	c2 (Social constant)	2
	w (Inertia constant)	0.6
MFO [15]	b(Logarithmic spiral)	1
	r (convergence constant)	linearly decreased from -1 to -2

**Table 3:** Results of unimodal benchmark functions.

Functions	Statistics	LFMVO	MVO	PSO	MFO
f1	Best	3.3178e-006	0.6989	1.5809e-005	2.2253
	Mean	8.6397e-006	1.4317	9.6071e-005	4.5093e+003
	STD	1.0386e-005	0.3460	5.9722e-005	7.1401e+003
	Rank	1	3	2	4
f2	Best	1.5898e-049	0.7038	0.0020	0.6584
	Mean	1.5107e-047	28.9175	0.0324	44.1369
	STD	4.2885e-047	52.3235	0.0329	29.2929
	Rank	1	3	2	4
f3	Best	1.4079e-005	152.5786	101.6546	9.8165e+003
	Mean	6.3973e-005	374.9805	177.3445	3.2987e+004
	STD	4.7714e-005	132.9893	53.1514	1.6263e+004
	Rank	1	3	2	4
f4	Best	8.1960e-004	0.8698	0.9602	51.6426
	Mean	0.0015	2.8432	1.4980	68.4824
	STD	4.6794e-004	1.3255	0.2266	6.6460
	Rank	1	3	2	4
f5	Best	38.7824	48.3483	34.5072	549.6379
	Mean	38.9045	621.7285	115.1391	2.0279e+006
	STD	0.0406	771.9059	74.2353	1.2651e+007
	Rank	1	3	2	4
f6	Best	8.2804	0.9215	6.6348e-006	3.4127
	Mean	8.6489	1.3681	1.0254e-004	4.5101e+003
	STD	0.1465	0.3078	9.8357e-005	7.1438e+003
	Rank	3	2	1	4
f7	Best	4.0043e-007	0.0169	0.0715	0.0999
	Mean	1.1291e-004	0.0367	0.2383	4.4739
	STD	1.0191e-004	0.0116	0.0829	8.4590
	Rank	1	2	3	4
Average Rank		1.28	2.71	2	4
Overall Rank		1	3	2	4

**Table 4:** Results of multi-modal benchmark functions.

Functions	Statistics	LFMVO	MVO	PSO	MFO
f8	Best	-1.7623e+004	-1.1953e+004	-4.7331e+003	-1.3585e+004
	Mean	-1.4886e+004	-1.0285e+004	-8.6352e+003	-1.1249e+004
	STD	5.4972e+003	792.9835	1.2979e+003	1.1557e+003
	Rank	1	3	4	2
f9	Best	0	107.0923	50.7752	117.6214
	Mean	0	171.9808	75.9237	217.1745
	STD	0	35.2283	14.5150	42.4181
	Rank	1	3	2	4
f10	Best	2.2204e-014	0.7559	0.0024	1.1029
	Mean	2.4558e-013	1.7515	0.0941	16.9435
	STD	6.2728e-013	0.5808	0.2946	5.8916
	Rank	1	3	2	4
f11	Best	2.5505e-007	0.6177	2.1519e-007	1.0064
	Mean	0.0172	0.8146	0.0060	43.9017
	STD	0.0056	0.0668	0.0080	67.4905
	Rank	2	3	1	4
f12	Best	7.3649e-008	0.0351	1.0481e-007	3.7687
	Mean	0.0008	2.3601	0.0019	1.9200e+007
	STD	0.0036	1.3190	0.0123	6.8287e+007
	Rank	1	3	2	4
f13	Best	0.0384	0.0953	2.6017e-006	17.2521
	Mean	0.0921	0.1809	0.0050	1.0252e+007
	STD	0.0417	0.0898	0.0082	6.4837e+007
	Rank	2	3	1	4
Average Rank		1.33	3	2	3.66
Overall Rank		1	3	2	4

**Table 5:** Results of fixed-dimension multi-modal benchmark functions.

Functions	Statistics	LFMVO	MVO	PSO	MFO
f14	Best	0.9980	0.9980	0.9980	0.9980
	Mean	0.9980	0.9980	1.6429	1.4923
	STD	0	0	0.9107	1.2829
	Rank	1	1	3	2
f15	Best	3.3355e-004	3.0828e-004	3.0803e-004	5.7996e-004
	Mean	2.4005e-004	5.2835e-004	8.0481e-004	9.4639e-004
	STD	7.3980e-004	0.0117	2.0810e-004	3.7484e-004
	Rank	1	2	3	4
f16	Best	-1.0316	-1.0316	-1.0316	-1.0316
	Mean	-1.0316	-1.0316	-1.0316	-1.0316
	STD	0	0	0	0
	Rank	1	1	1	1
f17	Best	0.39789	0.39789	0.39789	0.3979
	Mean	0.39789	0.39789	0.39789	0.3979
	STD	0	0	0	0
	Rank	1	1	1	1
f18	Best	3.0000	3.0000	3.0000	3.0000
	Mean	3.0000	3.0000	3.0000	3.0000
	STD	0	0	0	0
	Rank	1	1	1	1
f19	Best	-3.8628	-3.8628	-3.8628	-3.8628
	Mean	-3.8628	-3.8628	-3.8628	-3.8628
	STD	0	0	0	0
	Rank	1	1	1	1
f20	Best	-3.3220	-3.3220	-3.3220	-3.3220
	Mean	-3.2619	-3.2675	-3.2655	-3.2249
	STD	0.0609	0.0610	0.0601	0.0557
	Rank	3	1	2	4
f21	Best	-10.1532	-10.1532	-10.1532	-10.1532
	Mean	-7.7833	-7.6859	-7.5651	-7.6339
	STD	2.5717	2.8304	2.8225	3.0626
	Rank	1	2	4	3
f22	Best	-10.4029	-10.4029	-10.4029	-10.4029
	Mean	-9.8284	-8.2990	-9.2505	-8.2915
	STD	2.8397	2.7932	2.3655	3.2963
	Rank	1	3	2	4
f23	Best	-10.5364	-10.5364	-10.5364	-10.5364
	Mean	-10.3201	-8.7133	-10.3335	-9.5119
	STD	2.2845	2.8921	1.2830	2.5077
	Rank	2	4	1	3
Average Rank		1.30	1.70	1.90	2.30
Overall Rank		1	2	3	4

### Results analysis of unimodal test functions

Since a unimodal benchmark function has one global optimum, it is suitable for benchmarking the convergence rates (exploitation) of algorithms. Table 3 lists the results of the benchmark functions f1-f7 for different algorithms. First, we rank the algorithm from the smallest mean solution to the largest mean solution. Then, we calculate the average rank with respect to these seven functions and determine the overall rank, as shown in Table 3. From the rank of each function, we see that the LFMVO results are superior to the other algorithms except for f6 where the PSO is better. However, LFMVO obtains the overall best rank. The experimental results show that the proposed algorithm has superior performance in terms of exploitation.

### Results analysis on multi-modal test functions

The multi-modal test function has a global optimum, and the number of local optima increases exponentially with the dimensions. It is suitable for benchmarking the exploration of algorithms. Table 4 lists the results of benchmark functions f8–f13 for different algorithms. We also adopt the rank scheme used in the previous sub-section. From the rank of each function, we can determine that the LFMVO are superior to those of other algorithms except for f11 and f13, where the PSO outperforms the LFMVO algorithm. Nevertheless, the LFMVO ranks best overall. The experimental results demonstrate that the performance of the LFMVO is highly competitive with respect to exploration and escape from poor local optima.

### Results analysis on fixed-dimension multimodal benchmark functions

Compared with functions f8-f13, functions f14-f23 are simpler due to their lower dimension and fewer local minima. The results of benchmark functions f14-f23 for different algorithms are shown in Table 5. Though most algorithms were able to easily reach optima for functions f14-f23, we still rank these algorithms. Each of the algorithms can find the optimum at the best condition. For functions f16-f19, there are no differences among the approaches. From Table 6, we find that the LFMVO reaches better solutions than other algorithms.

**Table 6:** Basic Information of Benchmark Circuits.

Benchmark	Number of Cores
d695	10
p22810	28
p93791	32

### Convergence analysis

To investigate the convergence behavior of the proposed algorithm, we compare the convergence curves of the LFMVO, MVO, PSO and MFO for four test functions.

**Fig 1:**
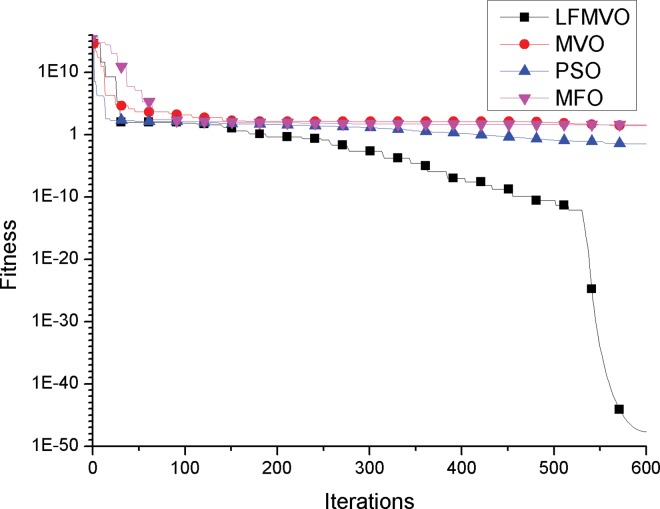
Sample graphs for convergence process comparison of LFMVO, MVO, PSO, and MFO over function f1.

**Fig 2:**
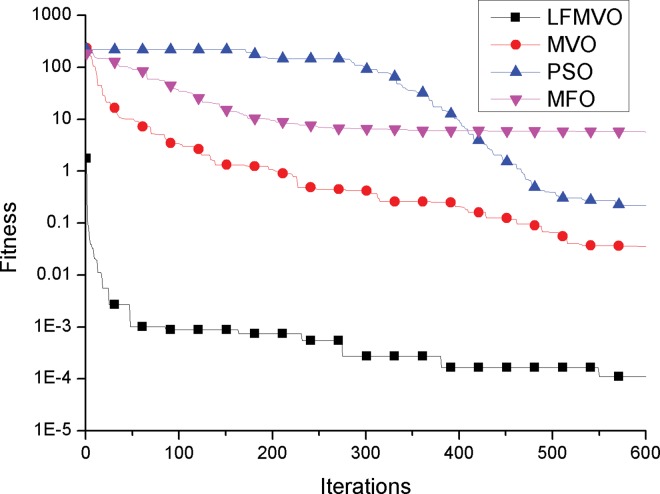
Sample graphs for convergence process comparison of LFMVO, MVO, PSO, and MFO over function f7.

The convergence curves of functions f2 and f7 (unimodal test functions) are illustrated in Fig 1 and Fig 2. The convergence curves show that the LFMVO algorithm can successfully improve the fitness of all universes and find a better solution over the course of iterations. The results of f2 and f7 demonstrate that the proposed algorithm has a very fast convergence speed. The reason is that objects in the universes with high inflation rates incline to shift to the universes with low inflation rates through white/black holes, so the fitness of all universes is get better over the course of iterations. Moreover, the proposed Levy flights phase can produce universes with long jumps that leads to quick convergence toward hopeful areas of the search spaces.

**Fig 3:**
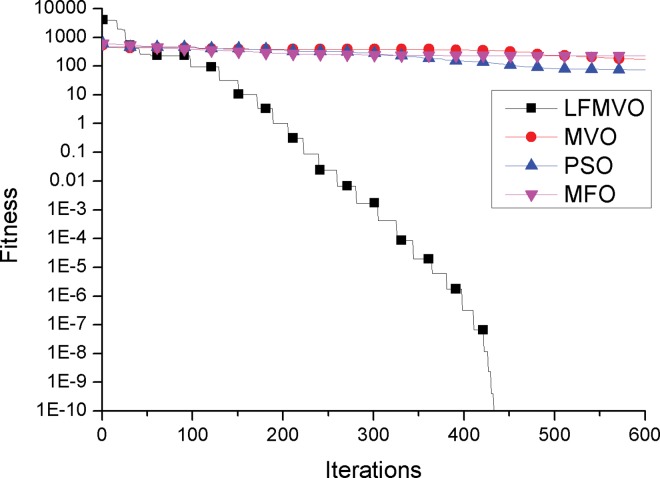
Sample graphs for convergence process comparison of LFMVO, MVO, PSO, and MFO over function f9.

**Fig 4:**
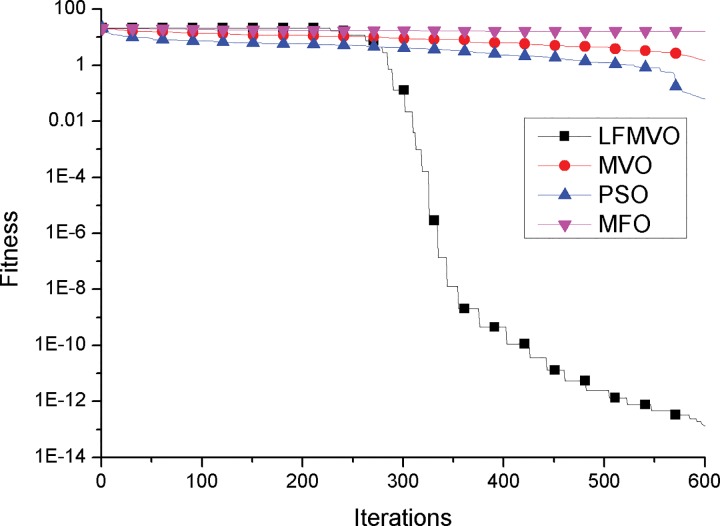
Sample graphs for convergence process comparison of LFMVO, MVO, PSO, and MFO over function f10.

The convergence curves of functions f9 and f10 (multimodal test functions) are illustrated in Fig 3 and Fig 4. The graphical results of f9 and f10 (multimodal test functions) show the superior local optima avoidance and global search ability of the LFMVO algorithm. The reason is that regardless of inflation rate, wormholes incline to exist stochastically in any universe which drive universes maintain the diversity over the course of iterations. In addition, Levy flights stage has the ability to escape from local optima and converge to the global optimum rapidly. We have provided the explanation between convergence and application in sub-section Convergence analysis.

The above results demonstrate the superior performance of the LFMVO algorithm in solving different benchmark functions compared with well-known algorithms. To further investigate the performance of the proposed LFMVO algorithm, a real engineering problem, which proved to be an NP complete problem, is solved in the following section.

## The application of the LFMVO on NoC test scheduling optimization

In this section, we apply the LFMVO to practical engineering applications to investigate the applicability and feasibility of the proposed algorithm. We estimate the performance of the LFMVO in terms of an engineering design problem—an NoC test scheduling problem. We describe the engineering applications generally and present the relevant mathematical models in the following paragraphs.

The NoC design paradigm has been proposed as an alternative to the traditional System-on-Chip (SoC) design paradigm for the next generation of complex Very Large Scale Integration (VLSI) [30]. The NoC is composed of IP cores, routers, resource interfaces and interconnection links. Due to the packet-switching network, the NoC provides high performance interconnection to embedded IP cores. However, testing embedded cores for NoC-based systems poses new challenges compared to traditional SoC [31].

Like traditional bus-based SoC, the general issues of the NoC system testing are composed of the test architecture design (test wrapper and TAM) and the test scheduling approaches. The test wrapper is the logic added around an embedded core, which is used to isolate the embedded core from surrounding logic and to offer test access to the core via a TAM. The TAM is the physical mechanism used to transport test stimuli and test responses for the cores. The test scheduling approach is employed to decide the test organization that targets test efficiency while considering all test constraints [32].

Testing is usually executed using automated test equipment (ATE), which offers test stimuli and estimates the test responses. ATE provides a limited number of tester pins (test channels) that can be used to send data to/receive data from the core-under-test (CUT). Inefficient use of tester pins (tester channels) has a negative impact on test cost [31].

In testing the embedded cores of NoC, we aim to minimize the test time while satisfying the test pins constraints and power constraints. The test time depends on the test architecture (test wrapper and TAM) and the corresponding test schedule with the test pins and power constraints. Here, we consider only the test wrapper proposed in [33] and a dedicated test access mechanism (TAM). The test scheduling problem for the NoC system can be defined as follows: in a NoC system, given the set parameters of cores Co, such that each core has a test time *T(c)* associated with the TAM width, the maximum test channels *Nt* for NoC, and the maximum power limit *PoL* for NoC, develop a test schedule, such that 1) *Nt* is not violated, 2) *PoL* is not violated, 3) the overall test time is minimized [18].

The embedded cores in a TAM are tested in series, and different TAMs are tested in parallel. The total test time is the sum of all the maximum test times for all the TAMs that are tested in parallel.

We introduce binary variables *y*_*ij*_ (1 ≤ *i* ≤ *N* and 1 ≤ *j* ≤ *B*) that are used to determine the assignment of cores to TAMs in the NoC. Each core in the system must be assigned to exactly one TAM.

We can formulate this unity condition by *y*_*ij*_ defined in Eq (24) with the unity condition formulated in Eq (25).

$$y_{ij}=\{\begin{array}{l} 1,\;\text{if core i is assigned to TAM j}\\ 0,\;\text{otherwise} \end{array}$$ (24)

$$\sum_{j=1}^{B}y_{ij}=1,\;1\leq i\leq N$$ (25)

The time needed to test all cores on TAM *j* is given by

$$\sum_{i=1}^{N}T_{i}(w_{j})y_{ij}$$ (26)

Since all the TAMs can be tested in parallel, the overall test time equals

$$T_{sum}=\max_{1\leq j\leq B}\sum_{i=1}^{N}T_{i}(w_{j})y_{ij}$$ (27)

The core test time is associated with the transmit bandwidth of the test data. Assuming that core *i* is assigned to TAM bandwidth *w*, the test time *T*_*i*_*(w*_*j*_*)* is defined by Eq (28):

$$T_{i}(w_{j})=\{1+\max(S_{in},S_{out})\}\times np+\min(S_{in}+S_{out})$$ (28)

where *S*_*in*_ *(S*_*out*_*)* denotes the length of the longest wrapper scan-in (scan-out) chain for a core and *np* denotes the number of test vectors. *T*_*i*_*(w*_*j*_*)* is calculated with a Best Fit Decreasing (BFD) algorithm for wrapper design from [31].

The total test pins used by the cores cannot exceed *Pin*_*max*_ during the whole test process. We can formulate the test pins used, $Pin_{used}^{t}$, during time slot *t* as follows:

$$Pin_{used}^{t}=\sum_{i=1}^{N}Pin_{i}\cdot \lambda_{i}^{t}\leq Pin_{\max}$$ (29)

where *Pin*_*max*_ is the total number of test pins available for testing.

$\lambda_{i}^{t}$ is defined by Eq (30):

$$\lambda_{i}^{t}=\{\begin{array}{c} 1,\;if\;TS_{i}\leq t\leq TE_{i}\\ 0,\;otherwise \end{array}$$ (30)

where *TS*_*i*_ and *TE*_*i*_ are the test start time and test end time of core *i*, respectively.

Although increasing the number of TAMs can effectively shorten the test time and reduce the test cost, it can lead to increasing test power. Therefore, to ensure the viability of the test, power must be constrained during the test.

In any test slot *t*, power consumption must satisfy

$$P_{m}^{t}=\sum_{i=1}^{N}P_{testi}\cdot \lambda_{i}^{t}\leq P_{\max}$$ (31)

where *P*_*testi*_ is the power consumption on core *i*, and *P*_*max*_ is the maximum power consumption allowed for the system.

Therefore, test scheduling for NoC can be formulated as follows:

$$\min T_{sum}=\max_{1\leq j\leq B}\sum_{i=1}^{N}T_{i}(w_{j})y_{ij}$$

$$s.t.\sum_{i=1}^{N}P_{testi}\cdot \lambda_{i}^{t}\leq P_{\max}$$

$$\sum_{i=1}^{N}Pin_{i}\cdot \lambda_{i}^{t}\leq Pin_{\max}$$ (32)

For the experiments, we used three SOCs from the ITC’02 SoC Test Benchmarks [34]: d695, p22810 and p93791 (see Table 6). We change the problem structure to use much bigger cases for the sake of observing convergence. In others words, we artificially constructed a hybrid system named hy629 including one d695, one p22810 and one p93791.

To compare conveniently, we used the same parameters as in the previous section. Every algorithm was run independently 40 times, and the best results of each algorithm for d695, p22810, p93791 and hyd629 are expressed in Tables 7–10, respectively.

**Table 7:** Experimental results for d695 with different test pins.

Pin_max_	P_max_	Test Time			
		LFMVO	MVO	PSO	MFO
256	100%	**9869**	**9869**	**9869**	**9869**
256	50%	**9869**	**9869**	**9869**	**9869**
256	30%	**13164**	**13164**	**13164**	**13164**
256	20%	**20163**	**20163**	20503	20528
192	100%	**12663**	**12663**	**12663**	**12663**
192	50%	**12663**	**12663**	**12663**	**12663**
192	30%	**13428**	**13428**	**13428**	**13428**
192	20%	**20188**	21022	21010	20751
128	100%	**18869**	**18869**	**18869**	**18869**
128	50%	**18869**	**18869**	**18869**	**18869**
128	30%	**18869**	**18869**	**18869**	**18869**
128	20%	**21401**	21989	21989	**21401**

**Table 8:** Experimental results for p22810 with different test pins.

Pin_max_	P_max_	Test Time			
		LFMVO	MVO	PSO	MFO
256	100%	**136400**	137436	138239	137586
256	50%	**135998**	137336	139132	138049
256	30%	**135907**	139229	139939	139388
256	20%	**136341**	140669	141498	139944
192	100%	**180942**	181393	181161	181283
192	50%	**180952**	181284	182496	181800
192	30%	**180954**	181510	184108	181995
192	20%	**181376**	181376	184111	182137
128	100%	**271331**	271360	271429	271343
128	50%	**271333**	271365	271529	271347
128	30%	**271332**	271407	271644	271356
128	20%	**271340**	271376	271655	271420

**Table 9:** Experimental results for p93791 with different test pins.

Pin_max_	P_max_	Test Time			
		LFMVO	MVO	PSO	MFO
256	100%	**306233**	310681	317253	312840
256	50%	**307278**	313360	342174	310842
256	30%	**368506**	373062	397351	387611
256	20%	**534687**	564496	567322	553547
192	100%	**407861**	410490	415412	410118
192	50%	**407856**	408154	408766	410896
192	30%	**408246**	415666	453412	422115
192	20%	**551352**	568489	577414	573920
128	100%	**611745**	611777	611762	611766
128	50%	**611748**	611766	611759	611801
128	30%	**611748**	611778	611774	611865
128	20%	**611789**	634631	658180	611974

**Table 10:** Experimental results for hybrid systems hyd629 with different test pins.

Pin_max_	P_max_	Test Time			
		LFMVO	MVO	PSO	MFO
512	100%	**243749**	261107	280109	271678
512	50%	**245305**	265048	282099	271730
512	30%	**246828**	264513	284344	284127
512	20%	**247289**	275809	285549	284513

**Fig 5:**
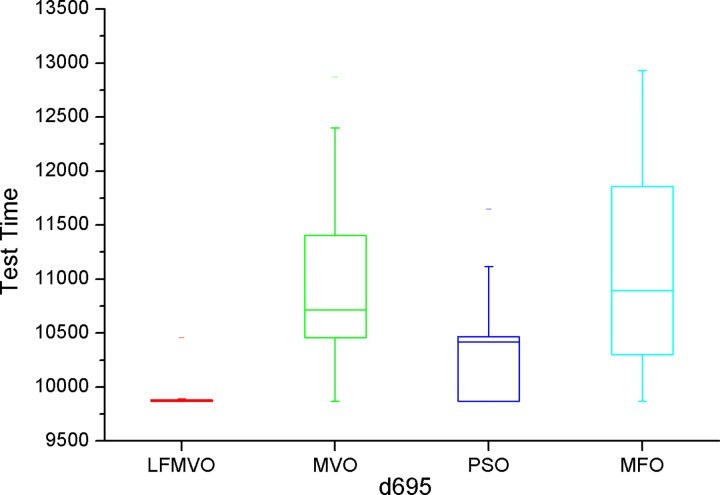
The boxplot of LFMVO, MVO, PSO, and MFO for d695.

**Fig 6:**
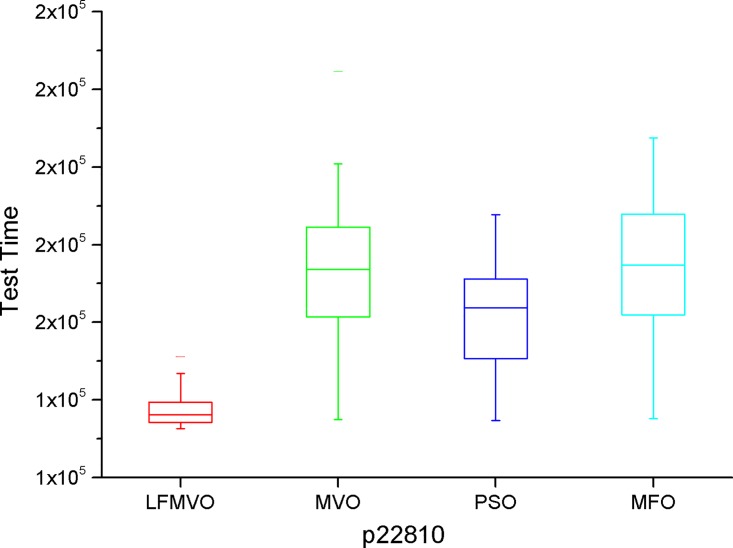
The boxplot of LFMVO, MVO, PSO, and MFO for p22810.

**Fig 7:**
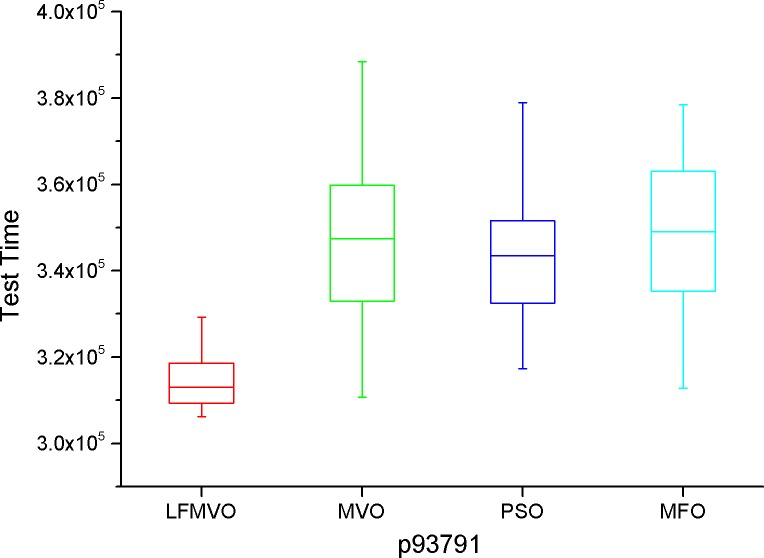
The boxplot of LFMVO, MVO, PSO, and MFO for p93791.

**Fig 8:**
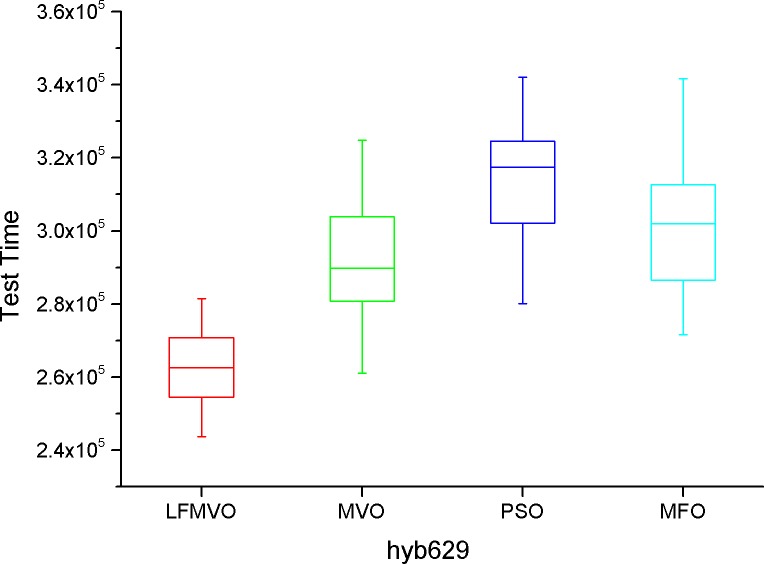
The boxplot of LFMVO, MVO, PSO, and MFO for hyb629.

The shortest test results times among the four algorithms are indicated by bold font for each method. From Table 7, we find that the four algorithms obtain the same results in most cases because d695 has the smallest scale among the three benchmarks. However, as the scale increases, the proposed algorithm yields the smaller test time in each category than the three reference methods. The experimental results of Tables 8–10 verify this statement. To further investigate the performance of the LFMVO (especially on the border/critical cases), we show the boxplots of the four algorithms for the different test benchmarks in Figs 5–8. Figs 5–7 show the condition Pin_max_ = 256 and P_max_ = 100%. Fig 8 shows the condition Pin_max_ = 512 and P_max_ = 100%. From Figs 5–8, we can see that the LFMVO outperforms other algorithms with respect to robustness and optimization accuracy. The reason of the superior results of LFMVO on application is that this proposed algorithm efficiently gains a balance between exploration and exploitation. For one, the concepts of white/black holes and Levy flights promote exploration, which can maximize the efficiency of resource searches in uncertain search space. For another, adding the existence of wormholes guarantees exploitation around the most hopeful area of the search space. In general, our proposed algorithm yields the higher performance in each statistical parameter than the three reference methods.

## Conclusions

A new algorithm named LFMVO is proposed in this paper, and it improves the performance of the MVO by incorporating Levy flights. The Levy flights component is introduced to enhance the global search ability of the MVO and its ability to escape from local optima. Experimental results on three sets of 23 well-known benchmark functions have verified that the proposed LFMVO has outstanding performance in speed of convergence and precision of the solution for global optimization in most cases. A real engineering application using NoC test scheduling optimization confirms that our proposed algorithm outperforms several state-of-the-art algorithms. This superior performance proves that the Levy flights are a promising way of strengthening the searching performance of MVO. Current studies implies that the LFMVO is a powerful and universal approach; it should be investigated further in several applications of engineering optimization problems, such as cloud computing, big data, smart city and vehicular named data networks [35–45]. Our future work is to extend the LFMVO to these fields.
